# Respiratory Responses during Therapeutic Positioning in Adults with and without Abdominal Obesity

**DOI:** 10.4314/ejhs.v35i4.5

**Published:** 2025-07

**Authors:** Ruchada Sriamad, Thaniya Klinsophon, Premtip Thaveeratitham

**Affiliations:** 1 Department of Physical Therapy, Faculty of Allied Health Sciences, Chulalongkorn University, Bangkok, Thailand

**Keywords:** Abdominal obesity, oxygen consumption, positioning, respiration, ventilation

## Abstract

**Background:**

Body positioning is commonly employed in hospitals and intensive care units to enhance respiratory function and minimize complications. While excess abdominal fat is known to affect respiratory mechanics, its impact across various body positions remains unclear.

**Methods:**

This study included 52 participants, categorized into normal weight and abdominal obesity groups. Respiratory parameters—including tidal volume (V_T_), minute ventilation (V_E_), oxygen consumption (VO_2_), carbon dioxide production (VCO_2_), end-tidal oxygen (PETO_2_), end-tidal carbon dioxide (PETCO_2_), and metabolic equivalent (MET)—were measured after 40 minutes in five different positions: Fowler's, right lateral, left lateral, supine, and prone. Measurements were obtained using a metabolic stress testing system. A two-way linear mixed-effects model was used to compare outcomes across body positions and participant groups.

**Results:**

Prone positioning resulted in the highest V_T_ and V_E_ in both the normal weight (450.6 ± 112.5 ml; 6.9 ± 1.2 l/min) and abdominal obesity groups (630.2 ± 172.9 ml; 9.3 ± 2.4 l/min) (p < 0.01). VO_2_ and VCO_2_ were also highest in the prone position for both the normal weight (207.8 ± 43.2 ml/min; 196.6 ± 36.8 ml/min) and abdominal obesity groups (264.8 ± 83.3 ml/min; 250.7 ± 75.9 ml/min) (p < 0.01). MET values peaked in the prone position across both groups. VO_2_ and VCO_2_ were significantly higher in the abdominal obesity group when in the prone position.

**Conclusions:**

The prone position was the most effective in improving ventilation and metabolic responses in both normal weight and abdominal obesity groups, followed by Fowler's. Elevated VO_2_ and VCO_2_ in individuals with abdominal obesity may reflect increased respiratory and skeletal muscle effort due to excess fat mass.

## Introduction

Abdominal obesity, also known as visceral obesity, is characterized by excessive fat accumulation in the abdominal cavity, particularly around internal organs, and is associated with an increased risk of respiratory illnesses (1). This adiposity in the thoracic and abdominal regions contributes to respiratory disorders such as obstructive sleep apnea syndrome and obesity hypoventilation syndrome (2, 3), which are linked to increased morbidity and mortality (4). In males, higher lipoprotein lipase activity in visceral adipose tissue—regulated by testosterone—leads to larger and more abundant visceral fat cells compared to females (5), potentially contributing to higher intensive care unit (ICU) admission rates among male patients (6).

The impact of abdominal obesity on respiratory function is multifactorial, involving both mechanical and inflammatory components (7). Obesity has well-documented effects on lung volumes, including reductions in expiratory reserve volume (ERV), functional residual capacity (FRC), and total lung capacity (TLC), primarily due to mechanical compression of the chest wall and diaphragm by excess adipose tissue (8–10). Clinical studies have demonstrated that obesity impairs respiratory function (3), reduces lung compliance and chest wall mobility (11–14), and contributes to airway narrowing and closure, leading to gas trapping and lung inhomogeneity (15). These abnormalities can alter ventilation and oxygenation—key physiological indicators for assessing health risks and predicting respiratory disorders.

Obstructive sleep apnea (OSA) and obesity hypoventilation syndrome (OHS) are particularly prevalent in individuals with abdominal obesity due to increased upper airway collapsibility and impaired ventilatory control (16). Furthermore, fat distribution plays a critical role in respiratory health. Individuals with morbid obesity and uniform fat distribution tend to experience fewer respiratory complications than those with a similar body mass index (BMI) but greater central adiposity. Abdominal obesity, in particular, imposes mechanical pressure on the diaphragm, restricting lung expansion and compromising pulmonary function (17).

Several studies have shown that body positioning affects ventilation and oxygenation. Upright postures generally increase tidal volume (V_T_) (18), and seated positions have been associated with enhanced alveolar ventilation (V_A_) (19). The higher V_A_ observed in the seated posture is largely attributed to increased V_T_, which may help offset elevated physiological dead space (2, 20). Oxygen consumption (VO_2_) also increases in upright positions due to greater energy expenditure (21–24).

More recently, prone positioning—including awake prone positioning—has garnered attention for its ability to improve ventilation and oxygenation. This position enhances lung mechanics by promoting more uniform ventilation, reducing atelectasis, and improving oxygenation through decreased lung compression and better perfusion. Initially considered a rescue therapy for severe acute respiratory distress syndrome (ARDS), prone positioning has become a primary lung-protective strategy, especially during the COVID-19 pandemic (25, 26).

Therapeutic positioning has demonstrated supportive effects on organ function, although not all responses are beneficial. Understanding respiratory responses across different body positions is essential for effective clinical management. However, prior studies have several limitations, including a focus on healthy individuals and a lack of comprehensive coverage of all therapeutic positions. Additionally, the applicability of these findings to individuals with abdominal obesity remains uncertain.

To address these gaps, this study examined respiratory responses to therapeutic positioning in individuals with and without abdominal obesity. Given the established effects of abdominal obesity on respiration, we hypothesized that participants with abdominal obesity would exhibit altered respiratory responses across various body positions compared to those of normal weight. Specifically, we anticipated that the prone position would enhance ventilation and oxygenation in both groups but require greater physiological effort in those with abdominal obesity due to increased fat burden on the respiratory muscles.

## Methods

**Study design and ethical considerations**: This cross-sectional study was conducted to compare respiratory responses between individuals of normal weight and those with abdominal obesity across different body positions. The study was approved by the Research Ethics Review Committee for research involving human participants, Group I, Chulalongkorn University (certificate of approval number: 186/2021). Written informed consent was obtained from all participants before data collection. The procedures were conducted in the Physical Therapy Laboratory, Faculty of Allied Health Sciences, Chulalongkorn University, under a controlled ambient temperature of 25°C to minimize environmental influences, given that obesity is known to impair autonomic regulation in response to temperature changes (27).

**Figure 1:**
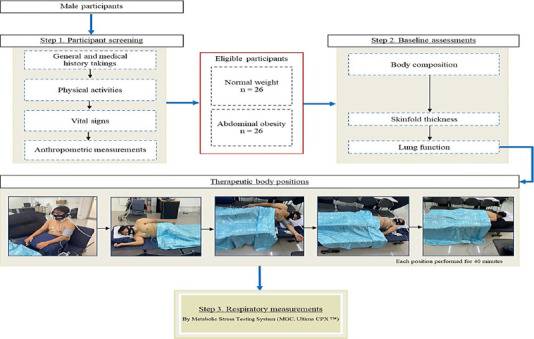
Research procedure

Participants were instructed to abstain from heavy meals for 4 hours and caffeine for 12 hours before testing. Each participant underwent screening, baseline assessments, and respiratory measurements after 40-minute positioning interventions, all completed on the same day (Figure 1).

**Participant screening**: First, general and medical histories were reviewed via questionnaire. Physical activity levels were assessed using the Baecke questionnaire (28). After a 10-minute rest, vital signs—including heart rate (HR), systolic blood pressure (SBP), and diastolic blood pressure (DBP)—were measured using an automatic digital blood pressure monitor (Yuwell, YE670D). Respiratory frequency (Rf) was recorded by counting breaths over one minute, and oxygen saturation (SaO_2_) was assessed using a fingertip pulse oximeter (ChoiceMMed™, MD300C1).

Anthropometric measurements included weight (via stadiometer), height (measured from floor to vertex), BMI [calculated as weight (kg)/height (m)^2^] (2), waist circumference (WC), and hip circumference. The waist-to-hip ratio (WHR) was then calculated.

**Baseline assessments**: Body composition was assessed using bioelectrical impedance analysis (BIA; Karada Scan, OMRON HBF-375) and skinfold thickness using a digital caliper (Digital Caliper Gauge, EDC-A1150). Skinfolds were measured three times and averaged at five anatomical sites in a standing position:

- Chest (diagonal fold between axilla and nipple)
- Mid-axillary (vertical line intersecting the xiphoid level)
- Subscapular (2 cm below the scapula's inferior angle)
- Suprailiac (above the iliac crest at mid-axillary line)
- Abdomen (5 cm right of the umbilicus).

Lung function was measured using spirometry (MGC, Ultima CPX™). Participants sat upright, wore a nose clip and mouthpiece, and performed a spirometric test: five tidal breaths, followed by maximal inhalation and forceful exhalation.

**Participants**: A pilot study with 10 male participants (5 normal weight, 5 abdominal obesity) estimated the effect size for respiration-related outcomes (effect size = 0.40; G*Power). Based on this, the full study recruited 52 participants to achieve 80% power (β = 0.20) at a = 0.05 for detecting group differences in minute ventilation (V_E_), the primary outcome.

**Inclusion criteria**: Males aged 30–59 years, Normal weight: BMI 18.5–22.9 kg/m^2^, WC < 90 cm, WHR ≤ 0.95, Abdominal obesity: BMI ≥ 25 kg/m^2^, WC ≥ 90 cm, WHR > 0.95

(Note: BMI criteria reflect Asian-specific cutoffs for obesity) (29–34)

**Exclusion criteria**: Diagnosed cardiovascular, respiratory (including OSA or OHS), renal, endocrine, or neurological conditions, use of medications affecting respiratory function, smoking, excessive alcohol intake, abnormal chest anatomy, or recent thoracic surgery

All participants had sedentary physical activity levels (Baecke score < 6), HR between 60–100 bpm, SBP < 140 mmHg, DBP < 90 mmHg, Rf 12–20 breaths/min, and SaO_2_ between 95–100%.

**Outcome measures**: After each 40-minute positioning intervention, respiratory variables were recorded over one minute using a metabolic stress testing system (MGC, Ultima CPX™). Participants breathed ambient air (21% oxygen) through a mask or mouthpiece with a pneumotachograph flow sensor. Calibration was performed before each session.

Parameters measured included R_f_, V_T_, V_E_, SaO_2_, FiO_2_, PETO_2_, PETCO_2_, VO_2_, VCO_2_, V_E_/VO_2_, V_E_/VCO_2_, respiratory quotient (RQ), and metabolic equivalent (MET). HR and BP were also monitored.

**Primary outcome**: V_E_, as a core measure of respiratory effort and function.

**Secondary outcomes**: All other variables listed above.

**Intervention**: Participants underwent all five body positions in a fixed sequence, from least to most compressive on the thoracic cavity:

**Fowler's**: Seated with 90° trunk inclination, hips flexed, knees slightly abducted and straight

**Right lateral**: Lying on right side, right leg straight, left leg supported with a pillow

**Left lateral**: Lying on left side, left leg straight, right leg supported with a pillow

**Supine**: Lying on back, head neutral, hips/knees slightly flexed and supported

**Prone**: Lying face down, head neutral, supported by a U-shaped pillow

**Data analysis**: Statistical analyses were performed using SPSS v22.0 (IBM Corp.). Normality was tested using the Shapiro–Wilk test and Q-Q plots. A two-way linear mixed-effects model assessed the effects of BMI group and body position on respiratory outcomes. Subjects were treated as random effects. Bonferroni post hoc tests were applied for pairwise comparisons. A *p*-value < 0.05 was considered statistically significant.

## Results

**Participant characteristics**: Table 1 presents the baseline characteristics of both groups. The mean age was 41 ± 8.55 years for the normal weight group and 41 ± 7.44 years for the abdominal obesity group. The latter exhibited significantly higher body weight, BMI, WC, HC, WHR, body fat, visceral fat, subcutaneous fat, and skinfold thickness across all sites (*p* < 0.001), underscoring marked differences in anthropometric and adiposity measures

**Table 1:** Participant characteristics

Characteristics	Mean ± SD		*p*-value

	Normal weight (N = 26)	Abdominal obesity (N = 26)	
Age (y).	41 ± 8.55.	41 ± 7.44.	0.385.
Body weight (kg).	63.0 ± 6.64.	80.02 ± 9.20.	< 0.001*
Height (cm)	169.3 ± 7.26.	168.73 ± 6.54.	0.379.
Body mass index; BMI (kg/m^2^).	21.9 ± 1.29.	28.07 ± 2.44.	< 0.001*
Waist circumference; WC	84.0± 4.78.	97.9 ± 5.42.	< 0.001*
Hip circumference; HC.	95.6 ± 4.85.	100.7 ± 4.58.	< 0.001*
Waist-to-hip ratio; WHR.	0.9 ± 0.05.	1.0 ± 0.03.	< 0.001*
Physical activity.	5.5 ± 0.42.	5.4 ± 0.49.	0.394.
Heart rate; HR (bpm).	72 ± 10.45.	75 ± 8.97.	0.133.
Systolic blood pressure; SBP (mmHg).	121 ± 10.32.	125 ± 10.18.	0.096.
Diastolic blood pressure; DBP (mmHg).	82 ± 9.02.	82 ± 7.35.	0.369.
Mean arterial pressure; MAP (mmHg).	95 ± 8.66.	97 ± 7.70.	0.220.
Respiratory frequency; R_f_ (bpm).	14 ± 3.40.	15 ± 2.83.	0.127.
Oxygen saturation; SaO_2_ (%).	98 ± 0.69.	9 ±0.89.	0.905.
Body compositions.	.	.	
Body fat (%).	22.05 ± 4.22.	27.35 ± 3.55.	< 0.001*
Visceral fat (%).	7.69 ± 3.07.	13.48 ± 2.67.	< 0.001*
Subcutaneous fat (%).	15.97 ± 4.19.	19.98 ± 3.81.	< 0.001*
Skinfold thickness.	.	.	.
Chest (mm).	10.49 ± 4.21.	17.38 ± 6.55.	< 0.001*
Mid axillary (mm).	8.65 ± 3.64.	16.97 ± 7.87.	< 0.001*
Subscapular (mm).	13.67 ± 5.00.	25.38 ± 7.72.	< 0.001*
Suprailiac (mm).	16.28 ± 6.58.	23.85 ± 7.81.	< 0.001*
Abdomen (mm).	16.71 ± 5.82.	27.56 ± 7.43.	< 0.001*
Sum skinfold thickness (mm).	65.80 ± 20.96.	111.14 ± 30.37.	< 0.001*
Lung functions			
Tidal volume; V_T_ (ml)	503.85 ± 162.78	490.46 ± 96.50	0.869
Slow vital capacity; SVC (L)	3.58 ± 0.84	3.83 ± 1.17	0.194
Expiratory reserve volume; ERV (L)	1.03 ± 0.32	1.14 ± 0.68	0.212
Inspiratory reserve volume; IRV (ml)	2.08 ± 0.64	2.24 ± 0.77	0.208
Inspiratory capacity; IC (L)	2.58 ± 0.62	2.73 ± 0.74	0.222
Vital capacity; VC (ml)	3.61 ± 0.83	3.87 ± 1.19	0.177
Force vital capacity; FVC (L)	4.05 ± 0.63	3.78 ± 0.65	0.197
Force expiratory volume in 1 second; FEV_1_ (L)	3.40 ± 0.55	3.14 ± 0.58	0.051
Force expiratory volume in 1 second per force vital capacity; FEV_1_/FVC	84.00 ± 4.66	83.04 ± 5.81	0.257
Forced expiratory flow between 25% and 75% of vital capacity; FEF 25-75% (1/s)	3.58 ± 1.10	3.43 ± 0.93	0.294
Maximum forced expiratory flow; FEF max (1/s)	6.49 ± 2.14	6.05 ± 2.04	0.228

Data are represented as mean ± SD. All characteristics were tested by independent samples t-test.

* *p* < 0.001 (significance difference between normal weight and abdominal obesity groups.

### Therapeutic positioning and respiratory responses

**Figure 2:**
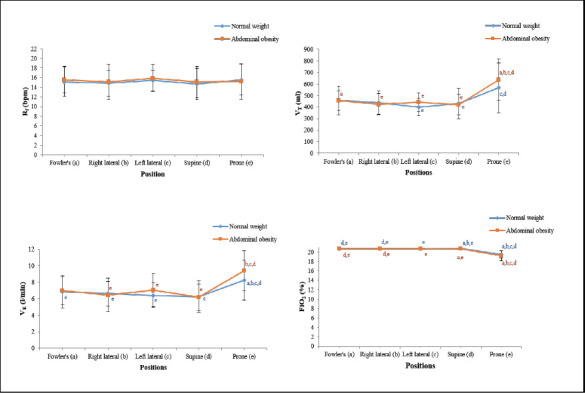
The influence of therapeutic positioning on ventilation. R_f_, respiratory frequency; V_T_, tidal volume; V_E_, minute ventilation; FiO_2_, fraction of inspired oxygen; (a) significance difference compared with Fowler's in the same group; (b) significance difference compared with right lateral in the same group; (c) significance difference compared with left lateral in the same group; (d) significance difference compared with supine in the same group; (e) significance difference compared with prone in the same group

**Ventilation**: No significant group differences were observed in R_f_. However, both groups exhibited significantly higher V_T_ and V_E_ in the prone position (*p* < 0.01). FiO_2_ was lowest in the prone position and highest in the supine position (*p* < 0.01). No significant group differences were detected in R_f_, V_T_, V_E_, or FiO_2_ within the same position (Figure 2).

**Figure 3:**
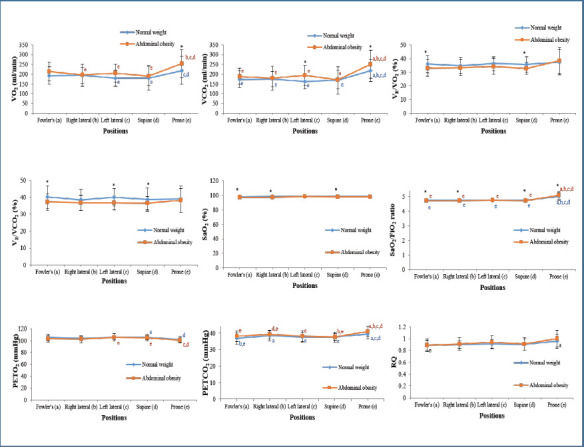
The influence of therapeutic positioning on oxygenation. SaO_2_, oxygen saturation; PETO_2_, end-tidal oxygen tension; PETCO_2_, end-tidal carbon dioxide tension VO_2_, oxygen consumption; VCO_2_, carbon dioxide production; V_E_/VO_2_, ventilator equivalent for oxygen; VE/VCO_2_, ventilatory equivalent for carbon dioxide; RQ, respiratory quotient; *, significant difference between normal weight and abdominal obesity in the same body position; (a) significance difference compared with Fowler's in the same group; (b) significance difference compared with right lateral in the same group; (c) significance difference compared with left lateral in the same group; (d) significance difference compared with supine in the same group; (e) significance difference compared with prone in the same group

**Oxygenation**: VO_2_, VCO_2_, and PETCO_2_ peaked in the prone position for both groups (*p* < 0.001; Figure 3). No positional differences were found for SaO_2_, V_E_/VO_2_, V_E_/VCO_2_, or RQ. However, group comparisons within positions showed significantly higher VO_2_ and VCO_2_ in the abdominal obesity group during prone positioning (*p* = 0.016 and *p* = 0.049, respectively). V_E_/VO_2_ was significantly lower in the abdominal obesity group in Fowler's and supine positions (*p* = 0.046 and *p* = 0.016). V_E_/VCO_2_ was also lower in the abdominal obesity group in Fowler's, supine, and left lateral positions (*p* = 0.035, *p* = 0.046, and *p* = 0.013). SaO_2_ was significantly lower in the abdominal obesity group in Fowler's, right lateral, and supine positions (*p* = 0.035, *p* = 0.021, and *p* = 0.041). No significant group differences were observed in PETO_2_, PETCO_2_, or RQ within the same position.

**Figure 4:**
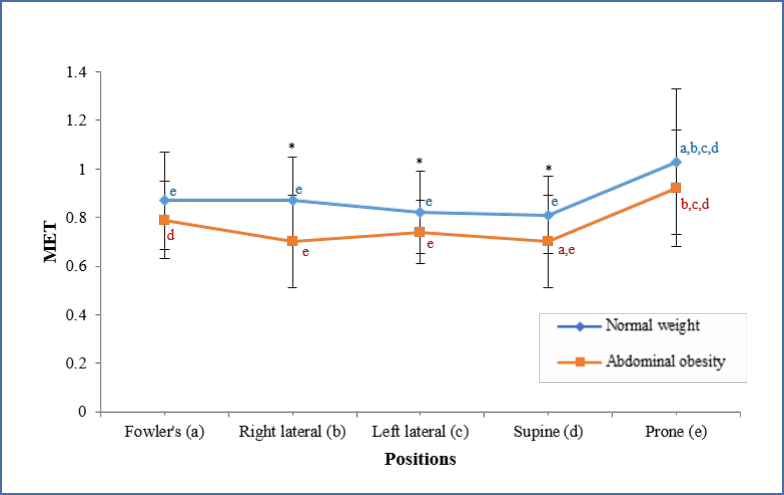
The influence of therapeutic positioning on metabolic equivalent. MET, metabolic equivalent; *, significant difference between normal weight and abdominal obesity in the same body position; (a) significance difference compared with Fowler's in the same group; (b) significance difference compared with right lateral in the same group; (c) significance difference compared with left lateral in the same group; (d) significance difference compared with supine in the same group; (e) significance difference compared with prone in the same group

**Metabolic Equivalent (MET)**: As shown in Figure 4, MET was significantly highest in the prone position and lowest in supine (*p* < 0.01). The abdominal obesity group exhibited significantly lower MET than the normal weight group in the right lateral, left lateral, and supine positions (*p* < 0.01, *p* = 0.039, and *p* = 0.013, respectively).

## Discussion

The results of this study demonstrate that body positioning significantly influences respiratory responses in individuals with abdominal obesity, with the prone position producing the most favorable outcomes in terms of ventilation, oxygen consumption, and carbon dioxide production. Specifically, the prone position yielded the highest values for tidal volume (V_T_), minute ventilation (V_E_), oxygen consumption (VO_2_), carbon dioxide production (VCO_2_), end-tidal carbon dioxide (PETCO_2_), and metabolic equivalent (MET) in both groups. Conversely, it resulted in the lowest values of fraction of inspired oxygen (FiO_2_) and end-tidal oxygen (PETO_2_).

Comparatively, the abdominal obesity group exhibited lower oxygen saturation (SaO_2_), ventilatory equivalents for oxygen (V_E_/VO_2_), and carbon dioxide (V_E_/VCO_2_) in Fowler's, side-lying (right and left lateral), and supine positions than the normal weight group. In contrast, during prone positioning, the abdominal obesity group displayed higher VO_2_ and VCO_2_, indicating an elevated metabolic and respiratory demand. Additionally, MET values were significantly lower in the abdominal obesity group in right lateral, left lateral, and supine positions, underscoring the physiological impact of excess abdominal adiposity on respiratory efficiency. These findings support prior research suggesting that gravitational forces acting on the lungs and adjacent thoracoabdominal structures profoundly affect respiratory mechanics and function (35).

The increased VO_2_ and VCO_2_ observed in the abdominal obesity group, particularly in the prone position, likely result from the increased fat mass placing a greater burden on respiratory and skeletal muscles, thereby elevating energy expenditure. Interestingly, no significant differences in lung volumes were found between groups. Although obesity is typically associated with reduced functional residual capacity (FRC) and expiratory reserve volume (ERV)—leading to ventilation-perfusion mismatch and airway closure, especially in the supine position (36)—this study measured lung function in the seated position. Furthermore, the obesity group had only mild obesity (BMI 25–29.9 kg/m^2^), possibly explaining the absence of significant differences in baseline pulmonary function.

The increased V_T_ and V_E_ in the prone position suggest enhanced lung recruitment and improved mechanics in this posture. These ventilatory improvements are likely due to increased chest wall elastance and posterior lung expansion, facilitating greater alveolar recruitment and increased airflow (37). While Fowler's position demonstrated the second-highest V_T_ and V_E_ values, the posterior chest wall may be restricted by the backrest, limiting expansion relative to the prone position. The low FiO_2_ values observed in the prone position align with the increased V_T_, as larger tidal volumes dilute the inspired oxygen fraction (38). The reverse trend was observed in the supine position, which generally restricts lung expansion due to gravitational and abdominal pressure.

No significant differences in ventilation were found between the two groups across positions, likely due to their similar lung function and mild obesity classification (33). Furthermore, all obese participants were free of comorbid conditions—particularly respiratory diseases—which may have further contributed to the lack of group differences in V_E_.

The prone position imposes pressure on the thoracic region, restricting chest wall movement and increasing respiratory effort. This necessitates higher oxygen uptake by respiratory muscles and increases muscular activity, contributing to elevated VO_2_ and subsequent VCO_2_ and PETCO_2_ levels (39–40). Additionally, the decrease in PETO_2_ may reflect increased oxygen extraction and utilization.

Although not statistically significant, Fowler's position also improved VO_2_ and VCO_2_ following prone positioning, likely due to increased postural muscle activity. The lower V_E_/VO_2_ and V_E_/VCO_2_ ratios observed in the abdominal obesity group in Fowler's, supine, and left lateral positions may reflect increased mechanical load and airway closure due to excess abdominal fat compressing lung parenchyma (40), leading to reduced lung capacities and impaired ventilation. This is consistent with the reduced SaO_2_ seen in these positions, as diminished alveolar ventilation compromises arterial oxygenation.

The elevated VO_2_ and VCO_2_ in the prone position among individuals with abdominal obesity suggest that excess weight imposes greater skeletal and respiratory muscle effort, increasing energy demands compared to normal-weight individuals.

The MET findings support this interpretation, as prone positioning induced the highest energy expenditure in both groups. Prone posture likely triggers greater respiratory effort and overall muscle activation, especially in the presence of abdominal obesity. While the prone position significantly enhances ventilation, its increased energy demands and logistical challenges—such as accommodating medical equipment—necessitate considering alternative positions for certain patients.

These results highlight the clinical utility of therapeutic positioning to enhance respiratory function, particularly in bedridden individuals or those with compromised gas exchange—regardless of body weight.

Several limitations should be acknowledged. First, the study included only adult males. Therapeutic positioning is used across diverse patient populations; thus, responses may differ by sex, age, or underlying health conditions. Future studies should include females, children, older adults, and patients with respiratory disorders or limited mobility.

Second, only a 90-degree Fowler's position was evaluated. In clinical practice, varying angles of trunk inclination are used. Further research should explore how different Fowler's angles influence respiratory outcomes.

Third, important respiratory mechanics such as inspiratory effort, closing volume, and functional residual capacity were not assessed during interventions. These parameters could provide valuable insight into lung strain and should be included in future investigations.

Therapeutic positioning is a fundamental non-pharmacological intervention employed in patient care. Positions such as prone and Fowler's significantly affect respiratory responses through gravitational influences on thoracoabdominal structures. This study found that prone positioning produced the greatest improvements in ventilation, oxygen uptake, and carbon dioxide elimination, followed by Fowler's. In individuals with abdominal obesity, the prone position also induced significantly higher oxygen consumption and carbon dioxide production than in normal-weight individuals—attributable to excess adipose tissue increasing respiratory workload.

These findings underscore the importance of individualized positioning strategies to optimize respiratory function, particularly in populations with abdominal obesity. Despite its physiological benefits, prone positioning may increase metabolic demand and pose practical challenges, suggesting a need for alternative positions when appropriate.
